# Deep brain stimulation of the periaqueductal gray releases endogenous opioids in humans

**DOI:** 10.1016/j.neuroimage.2016.08.038

**Published:** 2017-02-01

**Authors:** Hugh Sims-Williams, Julian C. Matthews, Peter S. Talbot, Sarah Love-Jones, Jonathan CW Brooks, Nikunj K. Patel, Anthony E. Pickering

**Affiliations:** a School of Physiology, Pharmacology & Neuroscience, Medical Sciences Building, University of Bristol, Bristol BS8 1TD, United Kingdom; b Department of Neurosurgery & Pain Medicine, North Bristol NHS Trust, Bristol BS10 5NB, United Kingdom; c Imaging Sciences, MAHSC, University of Manchester, M20 3LJ, United Kingdom; d Clinical Research Imaging Centre (CRiCBristol), University of Bristol, Bristol BS2 8DZ, United Kingdom; e Department of Anaesthesia, University Hospitals Bristol, Bristol BS2 8HW, United Kingdom

**Keywords:** DBS, deep brain stimulation, DPN, diprenorphine, PAG, periaqueductal gray, CMPf, centre median-parafasicular, Deep brain stimulation, Positron emission tomography, Pain, Endogenous opioid, Periaqueductal gray, De-afferentation

## Abstract

Deep brain stimulation (DBS) of the periaqueductal gray (PAG) is used in the treatment of severe refractory neuropathic pain. We tested the hypothesis that DBS releases endogenous opioids to exert its analgesic effect using [^11^C]diprenorphine (DPN) positron emission tomography (PET). Patients with de-afferentation pain (phantom limb pain or Anaesthesia Dolorosa (*n*=5)) who obtained long-lasting analgesic benefit from DBS were recruited. [^11^C]DPN and [^15^O]water PET scanning was performed in consecutive sessions; first without, and then with PAG stimulation. The regional cerebral tracer distribution and kinetics were quantified for the whole brain and brainstem. Analysis was performed on a voxel-wise basis using statistical parametric mapping (SPM) and also within brainstem regions of interest and correlated to the DBS-induced improvement in pain score and mood. Brain-wide analysis identified a single cluster of reduced [^11^C]DPN binding (15.5% reduction) in the caudal, dorsal PAG following DBS from effective electrodes located in rostral dorsal/lateral PAG. There was no evidence for an accompanying focal change in blood flow within the PAG. No correlation was found between the change in PAG [^11^C]DPN binding and the analgesic effect or the effect on mood (POMS_SV_) of DBS. The analgesic effect of DBS in these subjects was not altered by systemic administration of the opioid antagonist naloxone (400 ug). These findings indicate that DBS of the PAG does indeed release endogenous opioid peptides focally within the midbrain of these neuropathic pain patients but we are unable to further resolve the question of whether this release is responsible for the observed analgesic benefit.

## Highlights

• Sequential opioid-PET imaging study of deafferentation pain patients.
• All obtained analgesic benefit from deep brain stimulators (DBS) in periaqueductal grey (PAG).
• PET imaging with diprenorphine showed DBS reduced binding of the radioligand in the PAG.
• Change in binding consistent with DBS-evoked release of endogenous opioids.

## 1 Introduction

The mechanisms of action of deep brain stimulation (DBS) for all indications are under active investigation (Gradinaru et al., 2009, Kringelbach et al., 2007, Okun, 2014) and it is evident that different neural substrates are modulated depending on implantation target, stimulation parameters and disease aetiology. DBS of the periaqueductal gray (PAG) has been used for the treatment of pain for over fifty years (Duncan et al., 1991) and continues to find use for refractory syndromes such as de-afferentation pain (Bittar et al., 2005). It was introduced following the striking demonstration of electro-analgesia obtained from stimulation of the midbrain in rats (Reynolds, 1969). Further studies localised this action to the PAG, which was found to engage a descending inhibitory system to alter spinal processing of nociceptive inputs (Mayer and Liebeskind, 1974, Mayer et al., 1971).

This electro-analgesic action of PAG DBS in animal models was reversible with the opioid antagonist naloxone (Akil et al., 1976) – consistent with the high density of opioid receptor binding in the PAG (Kuhar et al., 1973). Similarly, early studies of PAG DBS in patients reported analgesic actions that were reversible with opioid antagonists (Hosobuchi et al., 1977). However, it is now apparent from animal studies that there are both opioidergic and non-opioidergic mechanisms of PAG analgesia (Bandler and Shipley, 1994, Heinricher et al., 2009). Similarly, carefully controlled and blinded studies in patients showed PAG electro-analgesia is not fully blocked with naloxone and does not show cross-tolerance to systemically administered opioids (Duncan et al., 1991, Young and Chambi, 1987). Furthermore, many of the patients benefitting from DBS have previously failed trials of systemic opioids. Therefore there is an unresolved question about the neurochemical basis of the beneficial effects of DBS of the PAG.

We have sought to address the question of mechanism of analgesic action using an opioid radioligand positron emission tomography (PET) strategy to study patients with implanted DBS systems for intractable de-afferentation pain. Previous opioid PET studies in healthy subjects have demonstrated that acute painful stimuli (Zubieta et al., 2001) decrease opioid receptor availability in pain processing areas of the brain. This is due to the release of endogenous opioids that compete with the radio-ligand. We have employed an analogous opioid PET approach using [^11^C]-diprenorphine ([^11^C]DPN) to test whether acute DBS of the PAG causes release of endogenous opioid peptides with a particular focus on the mid-brain and brainstem.

## 2 Materials and methods

### 2.1 Patients and study design

After approval from the NHS ethical committee (Ref. 12/SW/0255), North Bristol NHS Trust research and development (Ref. 2875) and Administration of Radioactive Substances Advisory Committee (Ref. 595/3586/29156 – Karl Herholz), the study was registered with the National Institute for Health Research Comprehensive Clinical Research Network (Ref. 13580).

We performed a sequential PET imaging study to examine the effect of DBS on opioid radiotracer binding to all opioid receptor subtypes (using [^11^C]DPN, a non-selective opioid receptor antagonist) and on blood flow (using [^15^O]water) in the brain and brainstem. The study tested the primary hypothesis that activation of DBS releases endogenous opioid at the site of stimulation in the PAG. The secondary hypotheses were that DBS would also release opioid peptides in brainstem regions believed to play a role in pain processing and modulation. We also sought correlations between regional opioid release in these areas and analgesic benefit / improvement in mood.

We planned to recruit five subjects from an existing cohort of patients (*n*=9) being treated with DBS for refractory de-afferentation pain. These patients had DBS systems already implanted following funding approval from the NHS exceptional clinical need panel, according to the criterion of lack of response to optimal conventional treatment and no sign of spontaneous recovery/improvement after two years following the initial referral to a secondary care pain service. All patients had electrodes (Medtronic 3387) implanted into the PAG on the contralateral side to their pain using a magnetic resonance imaging–directed method of targeting (Patel et al., 2007). Several subjects also had electrodes implanted into the centre median-parafasicular (CM-Pf) complex or the ventro-posterolateral nucleus of the thalamus. For further details of the time-course of the response to treatment of the Anaesthesia Dolorosa patients see Sims-Williams et al. (2016).

**Fig. 1:**
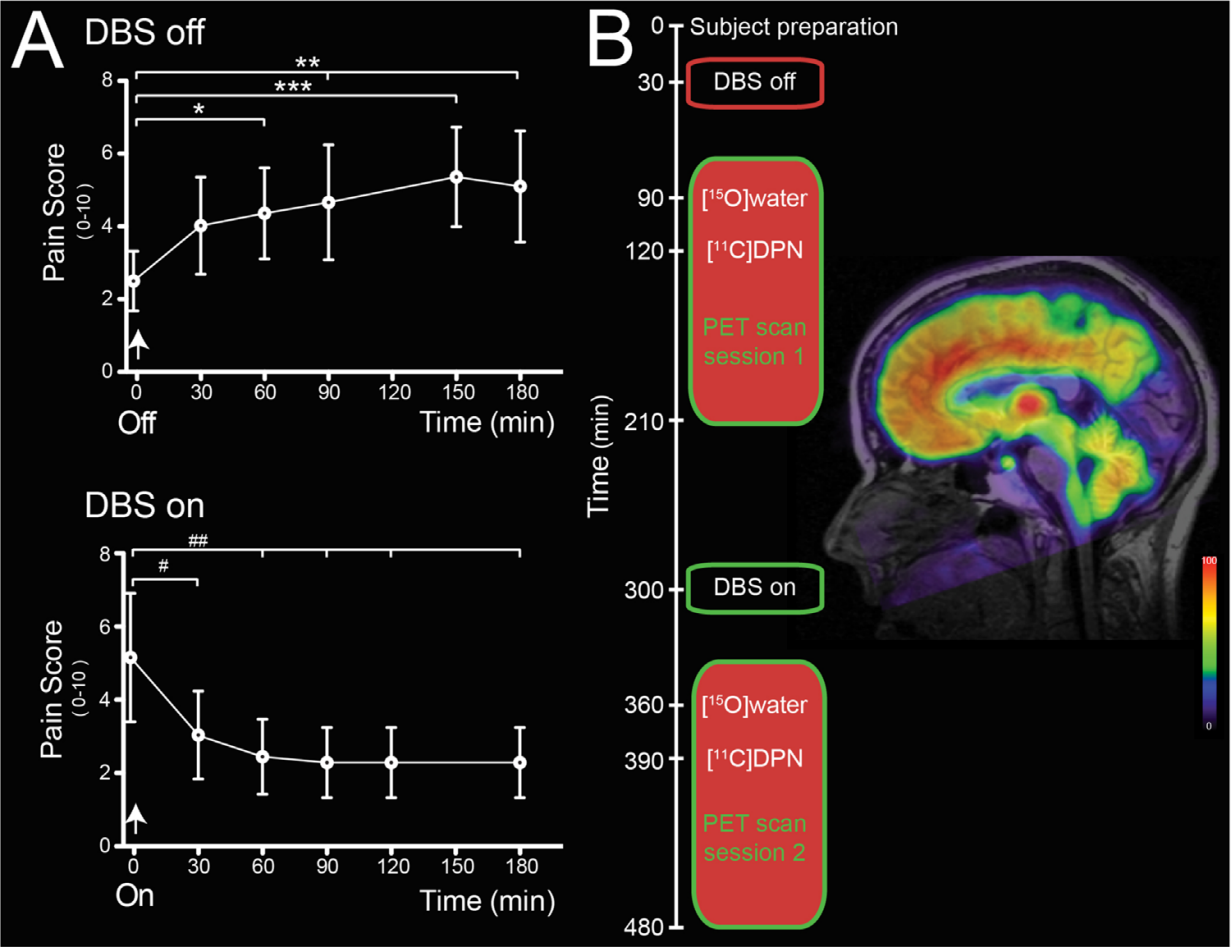
Acute analgesic effects of DBS and change in [^11^C]DPN binding. (A) Change in pain scores with DBS stimulation assessed during home trial. The analgesic effect of stimulation was almost completely lost 60 min after inactivation of DBS (pain VAS increased from 2.5±0.8 to 4.4±1.3, *p*<0.05, *n*=5) to reach a plateau level of pain that continued for more than two hours. On re-commencing PAG stimulation the analgesic effect was evident within 30 min (pain VAS reduced by 41% from 5.1±1.8 to 3.0±1.2) and this analgesic action was maintained for the rest of the test period. (rm-ANOVA with Dunnett's posthoc tests, *,# – *p*<0.05; **, ## – *p*<0.01, ***,### – *p*<0.001.). (B) Timeline of the sequential [^15^O]water and [^11^C]DPN scans with the first scanning session following 60 min after DBS was stopped and the second commencing 60 min after DBS was reinstated. Shown alongside is a representative sagittal parametric image of the volume of distribution (V_T_) for [^11^C]DPN overlaid on T_1_-weighted MRI image for a single subject (h00635) with DBS off. The image shows a characteristic distribution of the opioid ligand with high binding in the thalamus, midbrain and in several cortical regions (such as prefrontal and cingulate) but low binding in the occipital cortex and the pontine nucleus.

In designing the study we aimed to detect opioid release when the DBS system was activated and anticipated that this would be a different biological mechanism from the change seen in transitioning from DBS on to off where the process would be a dissociation of opioid from receptors and peptide degradation (hence with different kinetics). We employed kinetic modelling based on the arterial plasma [^11^C]DPN to maximise our sensitivity to detect changes in opioid binding. For ethical reasons we were mandated to minimise the number of cannulations (particularly relevant for our amputees) so performed the two scan sessions on the same day (allowing a single cannula). We performed sequential [^11^C]DPN and [^15^O]water scans with the first scan session with stimulator off followed by identical scans with stimulator on (protocol timeline in Fig. 1B). Subjects had >4 h interval between the sets of radiotracer injections to allow >5 [^11^C] half-lives (T_1/2_ 20 min) to minimise carry over effects due to radioactivity contamination of the second scan by the first scan dose. Study blinding was deemed unfeasible as all of our participants were aware of their stimulator being switched off because their pain returned.

Written informed consent was obtained from all subjects in accordance with the declaration of Helsinki. Eight subjects (three with Anaesthesia Dolorosa and five with Phantom Limb Pain) were screened for inclusion (criteria in Table 1). Two subjects were subsequently excluded; one due to poor acute temporal response to DBS activation/inactivation, the other could not tolerate stimulation withdrawal for sufficient time to allow PET scanning (~3 h). One subject withdrew from the study prior to PET scanning. The five enroled subjects, three with Anaesthesia Dolorosa and two with Phantom Limb Pain (Table 2, Inline Supplementary Clinical Vignettes), had their DBS stimulation settings reviewed and optimised where necessary prior to scanning (see Supplementary Table 1 for details).

An initial supervised trial “off stimulation” was performed in the clinic to introduce the pain and mood scoring scales. Subjects then completed a home diary, monitoring the effect of stimulation on visual analogue pain scores (VAS, 0–10) and mood (Profile of Mood State Shortened Version questionnaire (POMS_sv_) (Shacham, 1983)). Each subject recorded three baseline VAS pain scores and a single POMS_sv_ assessment before switching off their PAG DBS and recording their pain score at intervals (10, 20, 30, 60, 90, 120, 150 and 180 min). After 120 min, subjects completed the POMS_sv_ questionnaire before switching their DBS back on; repeating the VAS and POMS_sv_ rating at the same intervals.

### 2.2 PET scanning

Subjects attended the University of Manchester Wolfson Molecular Imaging Centre for PET scanning. Female subjects completed a screening questionnaire to exclude the possibility of pregnancy. Prior to scanning, and under local anaesthesia, an 18-gauge cannula was inserted into the antecubital vein for the injection of the radiotracer and a 22-gauge arterial cannula was inserted into the distal radial artery for blood sampling. For the phantom limb subjects the arterial and venous cannulae were sited in the preserved arm – for all other subjects they were inserted on opposite sides.

A High Resolution Research Tomograph (Siemens, Knoxville, TN) camera (de Jong et al., 2007) together with both [^15^O]water and [^11^C]DPN tracers were used to measure radiotracer delivery and opiate receptor binding, first with PAG DBS off and subsequently repeated with PAG DBS active. The scanning environment was kept consistent across subjects and between sessions. Subjects were positioned with their head well within the 25 cm axial field of view of the PET camera, which enabled simultaneous brainstem and brain measurements. Accurate repeat positioning between the two scanning periods was enabled by recording table height and translation as well as laser cross hair and facial contour alignment on a video monitor. Any head movement during scanning was monitored using an optical neuro-navigation system (Polaris VICRA^®^). In 4/5 subjects all radiotracer injections were done on the same day during two scanning periods and in one subject the second [^11^C]DPN injection occurred on the following day (due to a radio-synthesis failure for the planned second scan) with the same protocol timings. Two subjects kept CM-Pf stimulation on throughout the PET scanning protocol to maintain control of their symptoms (h00634 and h00635). CM-Pf stimulation was switched off for a 12 h period prior to PET scanning in two subjects (h00631 and h00683) and was then left off throughout the session; the remaining subject (h00636) did not have a second functioning stimulator. Pain, mood and anxiety scores were taken at baseline, after DBS was switched off/on (90 min prior to the scan) and at 0, 30 and 90 min during each [^11^C]DPN scan.

#### 2.2.1 Scan data acquisition

Each of the two scanning sessions consisted of the same sequence of events. At the start of each session a Cesium-137 transmission scan was performed and used to correct the subsequent scans for photon attenuation and scatter (Knoess et al., 2004). This was followed by two separate sequential injections of [^15^O]water (596 [517-639] MBq, median [range]) using an automated injection system (Radiowater Generator, Hidex Oy), followed by an injection of [^11^C]DPN (radiochemical purity >95%, 507 [277–566] MBq, 2.5 [0.5–18.5] µg, median [range]) given intravenously over 20 s followed by a saline flush. Following injection of [^15^O]water a period of at least 15 min elapsed before any subsequent injection to allow for radioactive decay. PET data were collected over the entire duration of the three injections until 90 min postinjection of [^11^C]DPN. The PAG DBS system was switched off 60 min prior to the first [^15^O]water radiotracer injection (90 min prior to [^11^C]DPN) for the initial session and turned back on 4.5 h later and the sequence of radiotracer injections and scans was repeated.

#### 2.2.2 Arterial blood sampling and analysis

Prior to scanning a 12 mL baseline blood sample was taken to quality assure blood analytical measurements using [^11^C]DPN-spiked blood. Continuous arterial blood sampling was started (5 mL min^−1^) immediately prior to each [^15^O]water and [^11^C]DPN injection and continued until 6 (water) and 10 (DPN) minutes postinjection, with continuous radioactivity measurements made using a bespoke Bismuth Germanate detector (Ranicar et al., 1991). For the [^15^O]water injection discrete samples were taken at 4, 5 and 6 min postinjection with whole blood radioactivity concentrations determined and used to calibrate the continuous arterial blood measurements. For [^11^C]DPN, discrete samples were also taken at 5, 7.5, 10, 15, 20, 30, 40, 50, 60, 75, and 90 min postinjection with whole blood and plasma radioactivity concentrations determined. Blood radioactivity measurement were made using a bespoke calibrated NaI well counter. Additionally for the samples taken at 5, 10, 20, 30, 40, 60 and 75 min the fractional activity of un-metabolised [^11^C]DPN in blood plasma was determined with chemical separation performed using solid-phase extraction followed by High Performance Liquid Chromatography and with radioactivity measurements of the eluent made using a 10 well calibrated gamma counter (Wallac Wizard 1470, Perkin Elmer). A total of 47 mL (water) and 160 mL (DPN) of blood was withdrawn per radiotracer injection. From these measurements (after full corrections for background, dead-time and sample volume) input functions of [^15^O]water in arterial whole blood and [^11^C]DPN in arterial blood plasma were determined and used in the subsequent kinetic modelling.

#### 2.2.3 Image reconstruction

Image reconstruction was performed using the HRRT user community software implementation of a 3D re-projection algorithm (3DRP) (Kinahan and Rogers, 1989) and ordered subset expectation maximisation algorithm (OSEM) (Hudson and Larkin, 1994). Dynamic image volumes were reconstructed using 3DRP to measure changing radioactivity concentrations over a 6 min period following [^15^O]water administration with contiguous frame durations of 60 s (background), 10×5 s, 6×10 s, 3×20 s and 6×30 s. Similarly following [^11^C]DPN administration, image volumes were reconstructed using 3DRP over a 90 min period with contiguous frame durations of 60 s (background), 3×10 s, 7×30 s, 12×120 s, 6×300 s and 3×600 s. This image data was used for subsequent kinetic modelling. In addition, a single image volume of the activity concentration over the 90 s period following [^15^O]water injection was reconstructed using 3DRP. Likewise a single image volume of the activity concentration during the 90 min period following [^11^C]DPN injection was reconstructed using OSEM (16 subsets, 12 iterations) and using resolution modelling (HRRT user community default kernel (Comtat et al., 2008)). These later static images were used for the determination of spatial processing parameters using SPM. In addition the 90 s images following [^15^O]water injection were used to examine changes in regional blood flow. Corrections for photon attenuation, scatter, detector dead-time, and different lines of response sensitivities (normalisation correction) were applied for both sets of reconstructions. The scan of one subject had significant discrete motion; therefore reconstruction with motion correction was used which included motion based reframing of the data and corrections for photon attenuation and scatter (Segobin et al., 2009).

### 2.3 Data analysis

#### 2.3.1 Generation of parametric maps

Kinetic modelling was performed in order to generate parametric images. For [^15^O]water data the dynamic images were first convolved with a 4 mm full width at half the maximum (FWHM) Gaussian kernel with regional cerebral blood flow (rCBF) values subsequently estimated using the generalised linear least-squares (GLLS) method (Walker et al., 2012). For the [^11^C]DPN data, parametric images of the rate of [^11^C]DPN delivery (K_1_) and steady state total volume of distribution (V_T_) were estimated using spectral analysis modelling (Cunningham and Jones, 1993). The spectral analysis model was constructed using 100 spectra logarithmically spaced from 2×10^−2^ s^−1^ to 1×10^−3·2^ s^−1^ together with and additional spectra for blood volume which is similar to previously published methods (Hammers et al., 2007), and fitted using a non-negative linear least squared algorithm (Lawson and Hanson, 1974). For both tracers global delays between the blood and brain measurements were determined using TAC data from a region over the entire PET camera field of view and repeatedly fitting the models with different delays and using a golden ratio line search algorithm. For [^15^O]water an additional global dispersion of *τ*=10 s was used (Iida et al., 1986). The parametric images for both tracers were subsequently median filtered (3 iterations of 3×3×3 cubic kernel), as noise in the PET images results in a skewed non-Gaussian distribution of the estimated rate constants with a long high valued tail. In the case of [^11^C]DPN the skewedness was exacerbated by the wide spectral range as low as 1×10^−3·2^ s^−1^, which investigation showed was necessary despite the resulting increase in variance (Hammers et al., 2007).

#### 2.3.2 Manual region of interest delineation

Brainstem regions of interest were defined on a subject-by-subject basis from their T_1_-weighted MRI scans by reference to a human brainstem atlas (Naidich et al., 2009). The ROI masks included: *medulla* – from the top of the dens to the ponto-medullary sulcus; *pons* - continuing rostrally above the medulla to the ponto-mesencephalic sulcus; *midbrain* - ascending to the inferior border of the optic tracts; and within the midbrain the *PAG* corresponding to the grey matter columns surrounding the aqueduct.

#### 2.3.3 Spatial processing

Spatial processing of the data was performed using statistical parametric mapping (SPM12, Functional Imaging Laboratory, UCL). Firstly the two [^15^O]water scans for each of the two scanning periods were realigned to each other and subsequently registered together with the [^11^C]DPN data to the patient's pre-DBS implantation “planning” T1 MRI data using a rigid body transformation and a normalised mutual information cost function. For two subjects these pre-DBS MRI scans were limited to slices of the target and planned trajectory, which were insufficient for the study purpose. Therefore, we obtained further MRI images with the DBS electrode in situ according to the manufacturer's guidance (“*MRI GUIDELINES for Medtronic Deep Brain Stimulation Systems – 2010*”).

Spatial normalisation, the registration of the MRI data with an MNI template, was performed twice. Firstly, a symmetric T1 weighted MNI template image was created using a previously published approach (Didelot et al., 2010), and the MRI data registered to this template in order to transform the PET data, including estimated parametric maps of kinetic parameters, to this symmetric 2 mm MNI template using linear interpolation. This data was subsequently used for voxel-wise statistical analysis using SPM. Secondly, each patient's MRI data was registered to the default T_1_-weighted MNI template with the transformation inverse used to transform a region of interest atlas (Hammers et al., 2003) to each patient using nearest neighbour interpolation. A grey matter segmented image was then extracted from the MRI data, smoothed with a 3 mm FWHM Gaussian kernel to degrade it to the approximate resolution of the PET data and a threshold of half the full intensity applied in order to refine the atlas regions to voxels that are predominantly grey matter within each patient. These regions were subsequently transformed to each PET scan data, together with manually defined regions for the Medulla, Pons, Midbrain and PAG, and used to sample all dynamic images of radioactivity concentrations.

#### 2.3.4 Mapping of DBS electrode position to MNI space

For each subject the position of the DBS lead tip and the position of the active electrode site was identified from the planning MRI scan or repeat MRI scans with the stimulator in situ performed as part of the study. These co-ordinates were transformed into MNI space using the same individual subject spatial transformations (see Section 2.3.3). These were then projected onto the MNI space 0.5 mm template as 3 mm spheroids.

#### 2.3.5 SPM voxel-wise statistical analysis

Prior to statistical analysis, parametric images of rCBF, K_1_ and V_T_ were smoothed with a 2 mm FWHM Gaussian kernel in order to minimise the effect of small registration errors and functional differences between subjects. In addition, images of radioactivity concentration during the 90 s period postinjection of [^15^O]water were smoothed with a 6 mm FWHM Gaussian kernel. In order to detect any differential effects upon ipsilateral and contralateral brain regions in response to the unilateral site of PAG stimulation, two sets of images were included in the analysis, with the second set created by digitally swapping the left and right hemispheres for each patient and scan. This enabled statistical analysis of whether there are any contralateral to ipsilateral differences (lateralisation effects). A flexible fixed effects factorial design was used with a patient main effect (independent) and a side (stimulation or contra-lateral, not independent)×stimulation (on or off, not independent) interaction effect without grand mean scaling (all) and without intensity normalisation (V_T_) or using ANCOVA intensity normalisation (K_1_, [^15^O]water data). Contrasts were examined to compare: stimulation off versus on; lateralisation with stimulation off and on; and lateralisation with stimulation. A threshold of *p*<0.001 (uncorrected) was used for the resultant T maps with a cluster extent threshold of 20 voxels. With respect to the primary hypothesis a small volume correction was performed in SPM using a region consisting of the union of the individually defined subject PAG ROIs transformed into MNI space using nearest voxel interpolation.

#### 2.3.6 Analysis by region of interest

The mean radioactivity values for the individually defined brainstem regions of interest and the cerebellum (defined from the Hammers' atlas (2003)) were calculated to create time activity curves (TACs) for all [^15^O]water and [^11^C]DPN scans. Subsequently kinetic modelling was employed using the same approach as with the generation of parametric images in order to derive regional values for K_1_, V_T_ and rCBF. Using these regional values we undertook pairwise comparisons (before and after stimulation) for the PAG and also nearby regions (pons, medulla and cerebellum) to see how focal the effects of stimulation were on V_T_. We also performed correlation analysis of the DBS-induced change in V_T_ against change in pain VAS (Pearson's correlation) and POMS_SV_ (see Table 3, using total and subdomain scores with Spearman's rank correlation).

### 2.4 Double blind, cross-over trial of acute naloxone administration

Four of our five study participants were invited back to clinic for a follow up experiment to see if the beneficial effect of their DBS stimulation was sensitive to the opiate antagonist naloxone. One patient (h00635) was excluded because they were maintained on regular slow release morphine that would confound the interpretation of any naloxone effect. Participants had two testing sessions (each lasting one hour) in a single day where they had baseline assessment of spontaneous pain (VAS) and quantitative sensory testing (QST). The QST was conducted over their painful area and assayed heat and cold pain threshold (skin temperature ramped with a thermode from 32 °C at ±1.5 °C/s using a Medoc TSA-II, Israel) and dynamic allodynia (using a cotton bud brushed at ~3 cm s^−1^). Patents were randomised to receive either naloxone or saline (placebo) intravenously in a cross-over design. An incremental bolus dosing schedule was employed to administer a total of 400 micrograms of naloxone (in aliquots of 50, 100 and 250 micrograms at 3 min intervals) or an equivalent volume of saline over a 10 min period. Neither the investigator nor the subject knew the study drug allocation and a priori the dose escalation would terminate in the event that the patient reported a worsening of their pain of >30%. Immediately after each dose the patients had repeat VAS and then at regular intervals until 30 min had elapsed when they had repeat QST. A period of 2 h elapsed between each limb of the cross-over study.

## 3 Results

### 3.1 Analgesic action of DBS

We recruited five patients who had PAG DBS systems (implanted one to five years previously) for de-afferentation pain (see Table 2 and inline supplemental Clinical Vignettes). These patients all obtained >50% improvement in their pain following DBS system implantation along with a reduction in their analgesic medication (inline Supplemental Table 2). Importantly each still showed an acute “on–off” analgesic response to PAG stimulation (see Fig. 1A) with a significant improvement in mood with stimulation (74% reduction in their Profile of Mood State Shortened Version score (Shacham, 1983), *p*<0.01, Table 3). In each patient the stimulation-evoked analgesia was evident within 30 min of DBS activation (pain VAS decreased by 41% from 5.1±1.8 to 3.0±1.2, *p*<0.05) and was maintained for the next two hours. The analgesic effect of DBS was lost 60 min after DBS withdrawal (pain VAS increased from 2.5±0.8 to 4.4±1.3, *p*<0.05).

### 3.2 Opioid PET scanning

Based on this DBS profile of action each subject had initial [^11^C]DPN and [^15^O]water PET scans with their DBS off and then again after switching their DBS on. After reconstruction of the PET data with kinetic modelling we observed a characteristic pattern of opioid binding in the brain (Sprenger et al., 2005) (Fig. 1B).

### 3.3 DBS-evoked changes in opioid binding in the PAG

**Fig. 2:**
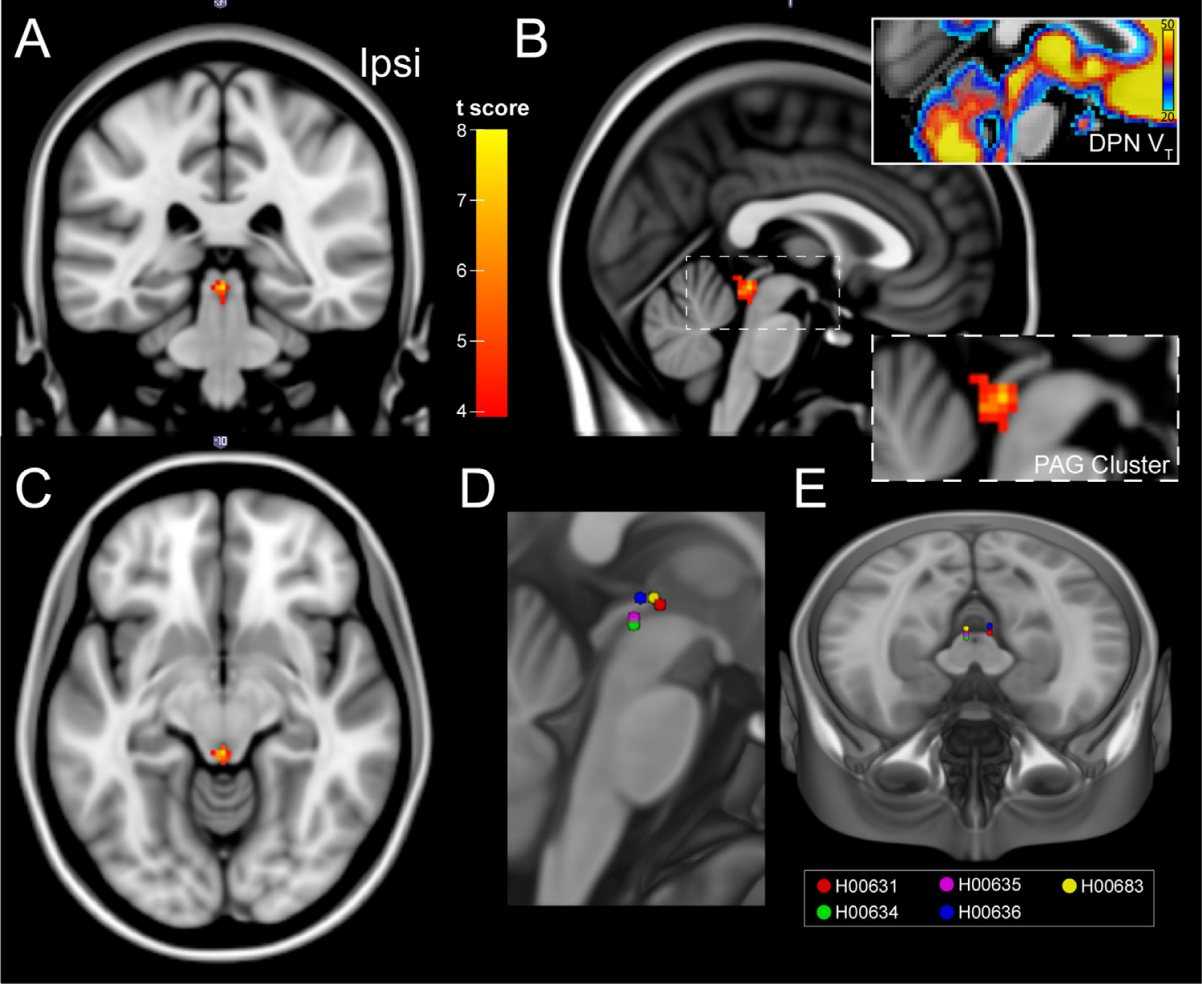
DBS reduces [^11^C]DPN binding in the PAG. (A–C) Brain-wide statistical parametric mapping (SPM) analysis across all patients showing changes in the total volume of distribution (V_T_) on DBS. The SPM analysis shows the T-map (see colour bar) for a single cluster (threshold *p*<0.001 (uncorrected); 20 voxel cluster extent) of 31 voxels located in the dorsal PAG (*p*=0.003 uncorrected) shown superimposed on MNI standard brain (1 mm) in coronal, sagittal and transaxial sections (ipsi – side of DBS). The inset panels in B show an enlarged image of the cluster and the equivalent sagittal section around the PAG with the superimposed parametric image of the group mean [^11^C]DPN V_T_ (DBS off condition) showing that the caudal PAG in the region of the cluster has the highest opioid binding. (D and E) For comparison the position of the DBS lead active cathode (centre of stimulation contact(s)) mapped into MNI space shown projected onto sagittal and horizontal sections. (For interpretation of the references to colour in this figure legend, the reader is referred to the web version of this article.)

**Fig. 3:**
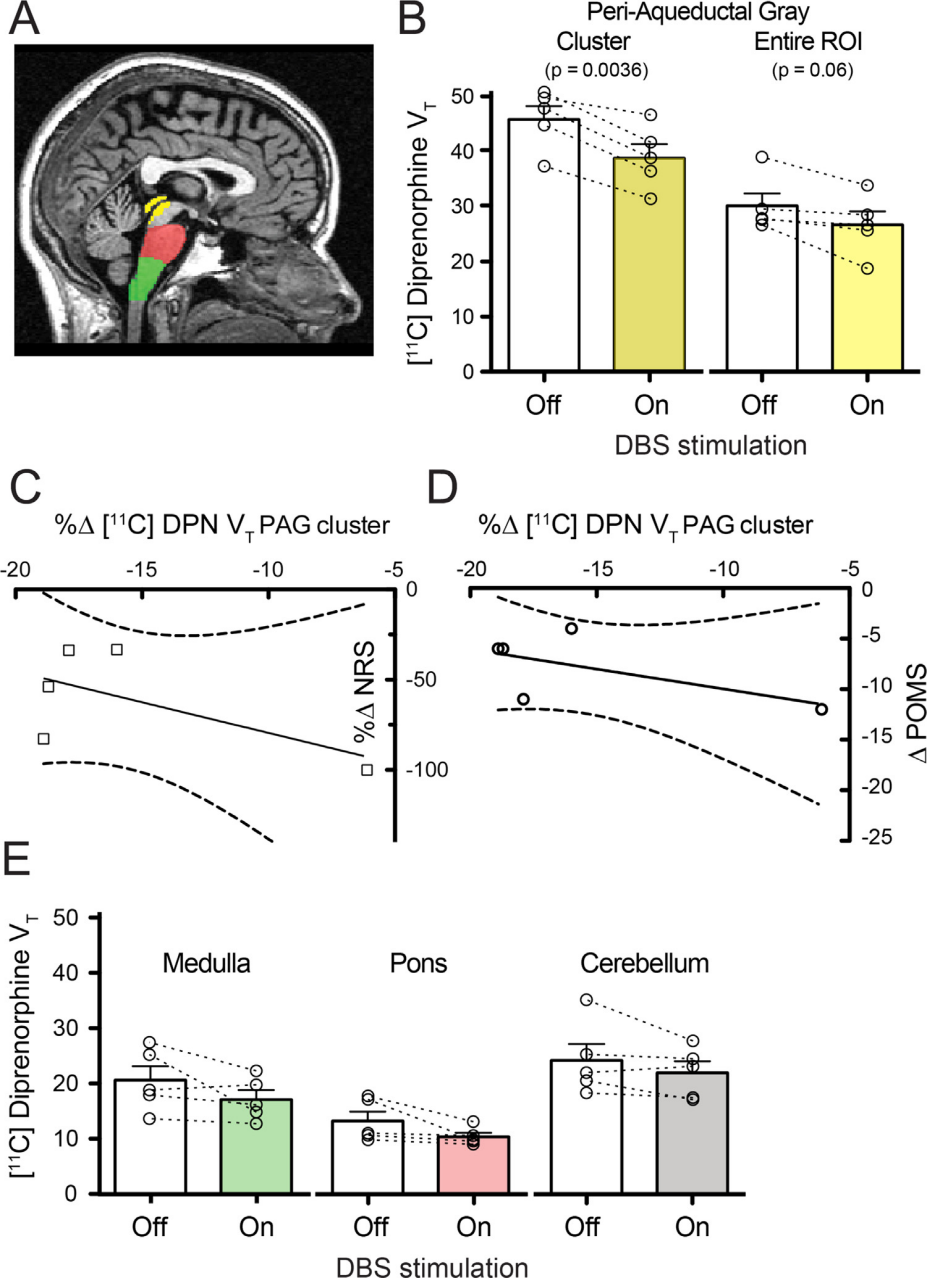
Region of interest analysis of changes in V_T_ [^11^C]DPN in the brainstem. (A) PAG (yellow), pontine (red) and medullary (green) regions of interest were defined for each subject allowing extraction of mean [^11^C]DPN V_T_. (B) Analysis of the PAG cluster and entire ROI showed a decrease in V_T_ with DBS for every patient that was significant at a group level for the cluster. (C and D) No correlation was found between the change in [^11^C]DPN V_T_ in the PAG cluster and either the pain NRS or the POMSsv (best fit linear regression lines shown with confidence intervals overlapping zero). (E) DBS did not produce a significant change in medullary, pontine or cerebellar [^11^C]DPN V_T_. (For interpretation of the references to colour in this figure legend, the reader is referred to the web version of this article.)

Brain-wide SPM analysis of V_T_ identified a single cluster of reduced [^11^C]DPN binding with DBS (see Fig. 2A–C, off–on contrast). This was located in the dorsal PAG with 31 contiguous voxels (*p*=0.002 after small volume FWE correction with the composite PAG mask) supporting the primary hypothesis that DBS reduced [^11^C]DPN binding through the release of endogenous opioids (no other clusters were found in this contrast or in the on–off or lateralised off–on contrasts). This PAG cluster was located at the caudal pole adjacent to the DBS stimulation electrode sites (Fig. 2D and E). Analysis of the PAG cluster showed that there was a fall in V_T_ of 15.5% with DBS (from 45.8±2.4 to 38.7±2.5, *p*<0.005, Fig. 3B). All subjects also showed similar falls in the whole PAG ROI V_T_ (Fig. 3B, *p*=0.06). No significant correlation was found between the DBS-induced reduction [^11^C]DPN V_T_ in the PAG cluster and the change in pain score or POMS_SV_ (shown in Fig. 3C and D, analysis for either absolute or proportionate change in each variable).

### 3.4 DBS-evoked changes in opioid binding in the brainstem

Given the known anatomical and functional connectivity between the PAG and the rostral-ventromedial medulla (Fields, 2004, Millan, 2002, Ossipov et al., 2010) (RVM) we performed an exploratory analysis of the change in [^11^C]DPN V_T_ in the medulla and the nearby pons and cerebellum. Although four of five subjects showed reductions in medullary [^11^C]DPN V_T_ (group average reduced by 17%, Fig. 3E) this did not achieve statistical significance (20.6±2.5 to 17.1±1.7, *p*=0.16, *n*=5, two tailed paired *t*-test). No significant change in [^11^C]DPN V_T_ was seen in the pons or cerebellum emphasising the regional specificity of the PAG changes (Fig. 3E).

### 3.5 DBS-induced changes in brain blood flow

To test whether the localised change in [^11^C]DPN V_T_ might be due to a focal change in blood flow with PAG DBS we performed a similar SPM analysis for: the K_1_ parametric maps (reflecting the rate of uptake of [^11^C]DPN from blood into tissue); for rCBF parametric maps; and for radioactivity concentrations during the 90 s period postinjection of [^15^O]water, as is commonly done when conducting activation studies. All of these analyses failed to show any relative changes in the vicinity of the PAG. This was further investigated using small volume correction with the PAG mask but again showed no evidence for localised changes with DBS in any of the three measures.

### 3.6 Lack of reversal of analgesic effect of DBS by naloxone

**Fig. 4:**
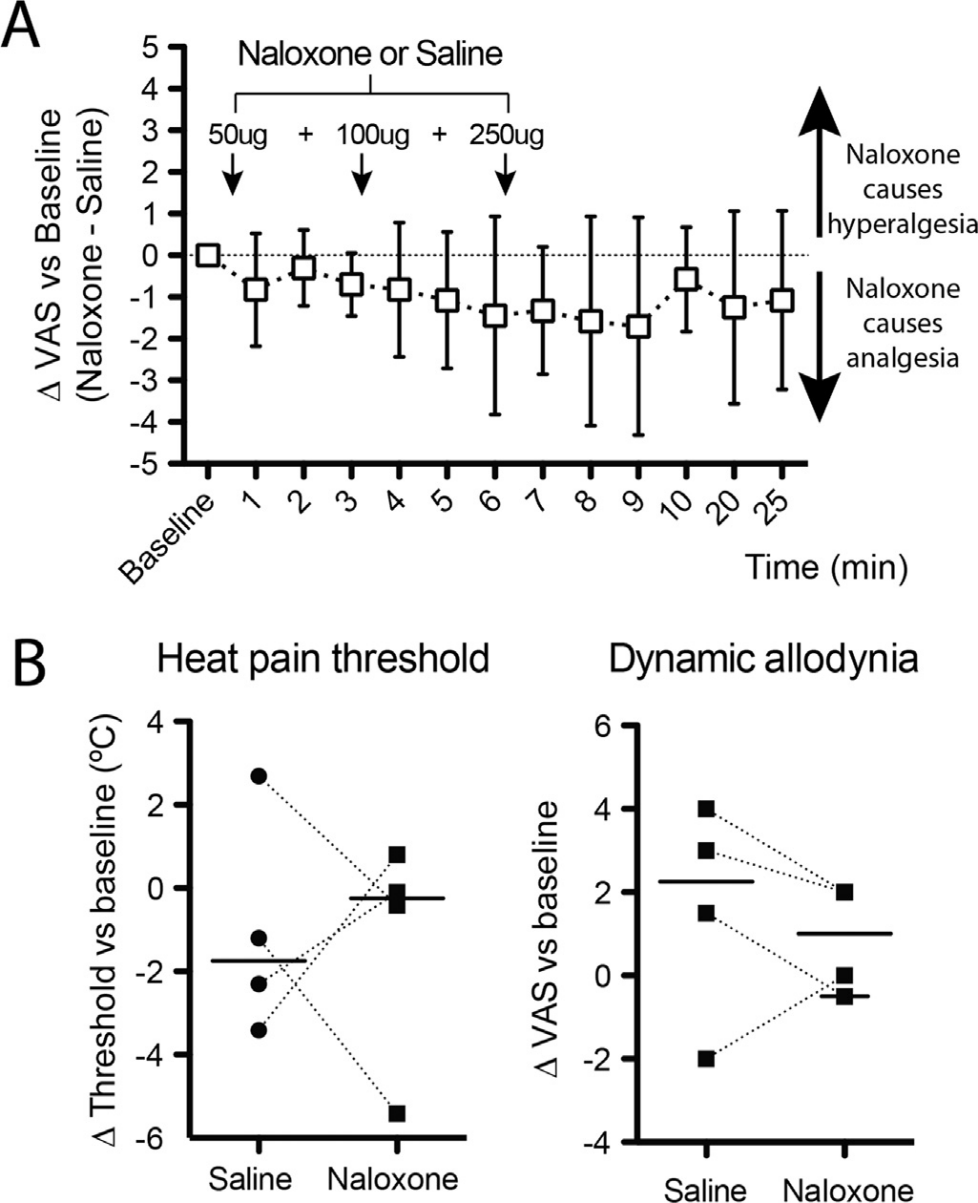
Naloxone does not change ongoing pain ratings or cause hyperalgesia. In a randomised, double blind, crossover design four of the DBS subjects were given either naloxone or saline and the effect on spontaneous and evoked pain assessed. (A) Naloxone did not produce hyperalgesia compared to saline alone. Graph shows the change in VAS (naloxone – saline) normalised to baseline VAS with positive values indicating that naloxone produced hyperalgesia (mean ±95%CI, One way rm-ANOVA, ns). (B) Assessment of the change from baselines in heat pain threshold and dynamic allodynia also showed no significant effect of naloxone on either measure. (lines indicate median values, paired *t*-test, ns).

Having demonstrated localised changes in opioid binding in the PAG with DBS, yet without any correlations with the analgesic or positive effect on mood, we wanted to further test whether this change in opioid binding was functionally meaningful. To do this we conducted a double blind, placebo controlled investigation on four of the five subjects (one excluded because they were taking regular opioid analgesia) and examined the effect of systemic naloxone (400 mcg), an opioid antagonist, on the DBS analgesic effect. The administration of naloxone to these subjects had no significant effect on ongoing pain scores or on evoked quantitative sensory test measures including heat and cold pain threshold and dynamic allodynia (see Fig. 4A and B).

## 4 Discussion

When it was first introduced for pain treatment, DBS was believed to trigger the release of endogenous opioids (Akil et al., 1976, Hosobuchi et al., 1977) which act upon opioid receptors in the PAG (Kuhar et al., 1973, Pert and Snyder, 1973) to produce the analgesic effect but this was subsequently questioned (Duncan et al., 1991, Young and Chambi, 1987). In this PET imaging study we show that DBS activation causes a focal reduction in [^11^C]DPN V_T_ in the PAG consistent with stimulation-evoked release of endogenous opioids. As well as having a high density of opioid receptors the PAG has intrinsic opioid peptide (enkephalin) synthesising neurons (Covenas et al., 2004), receives inputs from opioid containing terminals (see review Carrive, 2012) and opioid microinjection to the PAG produces potent analgesia (Yaksh and Rudy, 1978). Our findings therefore support the principle that DBS for pain is potentially acting through the endogenous opioid system.

Previous opioid PET studies have shown a reduction in binding of the µ-opioid radioligand [^11^C]carfentanil in the PAG during the application of noxious stimuli to volunteers (Wager et al., 2007, Zubieta et al., 2005) and also have shown a correlation between this reduction in [^11^C]carfentanil binding and the magnitude of placebo analgesia (Wager et al., 2007). This reduction of radioligand binding was proposed to represent competitive displacement by opioid peptides. Long-term changes in opioid binding (assayed with [^11^C]DPN) have also been found in the PAG in central neuropathic pain (Maarrawi et al., 2007a). The same group also showed a decrease in opioid binding in neuropathic pain patients after seven months of chronic motor cortex stimulation (Maarrawi et al., 2007b). DPN is a competitive, non-subtype selective opioid antagonist that has been used in binding studies in animal models that have shown it is displaced by opioid peptide ligands and also in response to activation of the endogenous opioid system (Neumaier and Chavkin, 1989, Ruiz-Gayo et al., 1992, Stein et al., 1990). Reduction of DPN binding has been reported in human volunteers after painful stimulation (Sprenger et al., 2006) and also in patients following reading-induced seizures (Koepp et al., 1998). Although DPN is less specific for mu opioid receptors and perhaps less sensitive than Carfentanil in detecting agonist evoked changes in binding (Quelch et al., 2014) this appears to have not been a limiting factor in detecting the DBS-evoked change in [^11^C]DPN V_T_. To our knowledge, ours is the first study to use a paired DPN scan design to resolve the acute reduction in opioid binding (within 90 min) triggered by DBS supporting the proposition that the reduction in [^11^C]DPN V_T_ is a consequence of competitive displacement by endogenous opioid (as proposed for Carfentanil (Zubieta et al., 2005) and DPN (Sprenger et al., 2006)).

The purpose of the kinetic modelling was to isolate changes in radioactivity concentrations due to the altered binding to opioid receptors (V_T_) from potential changes due to the radioactivity within blood and the delivery of this radioactivity to the brain regions. Despite this, inaccuracies in the model could have produced residual dependencies. Specifically, there is the potential for the regional reduction observed for V_T_ in the PAG to be due to regional changes in the delivery of the radiotracer (K_1_) which in turn is a consequence of regional changes in blood flow (rCBF) resulting from the electrical stimulation of brain parenchyma. To examine this we conducted similar voxel-wise statistical testing of derived [^11^C]DPN K_1_ and [^15^O]water rCBF parametric maps. We also conducted statistical testing using the radioactivity concentration during the 90 s following [^15^O]water injection which is commonly used as a surrogate for changes in rCBF. No significant changes were observed in the PAG region in all cases, showing that the observed changes in V_T_ within the PAG are not a consequence of any stimulation mediated changes in tracer delivery.

The discrete cluster of opioid displacement was located in a caudal dorsal/dorso-lateral territory of the PAG (Linnman et al., 2012) close to the sites of electrical stimulation (which mapped to the dorso-lateral and lateral areas of the rostral PAG/PVG). In rats and cats, the PAG is organised into columns from which specific patterns of autonomic, sensory and motor co-ordination can be evoked (Bandler and Shipley, 1994, Carrive, 2012, Lumb, 2002). Activation of the dorsal/dorso-lateral columns evokes responses characteristic of fight/flight behaviours with sympatho-activation, hypertension and a non-opioid mediated analgesia in animals. In contrast passive coping behaviours are triggered by stimulation of the ventro-lateral column to trigger hypotension and an opioid-mediated analgesia. There is supportive evidence from human studies indicating that DBS of the ventral PAG produces hypotensive actions (Green et al., 2007) and increases vagal tone (Pereira et al., 2010) whereas stimulation of the dorsal PAG increases blood pressure (Green et al., 2006). However, another study from the same team has suggested that changes in local field potentials produced by dorsal PAG stimulation were sensitive to naloxone (unlike ventral PAG) indicating that this involves opioids (Pereira et al., 2013). Recent evidence from human MR imaging studies suggests that a similar columnar organisation is present in man as seen in rodents (Coulombe et al., 2016, Ezra et al., 2015, Faull et al., 2015). However, there appear to be cross-species differences in anatomical connectivity in man particularly for the dorsal PAG that appears to have good brainstem connectivity consistent with a potential role in descending control of nociception (Ezra et al., 2015, Pereira et al., 2010). Our data suggests that the DBS-evoked opioid displacement was predominantly in the caudal and dorsal PAG although we acknowledge that the PET imaging methodology affords limited spatial resolution to discriminate between the relatively small columns. We also noted that the cluster was located over the area with the highest [^11^C]DPN V_T_ within the PAG suggesting that it has a high density of opioid receptors.

Investigations in animals have shown that the PAG regulates spinal nociceptive processing via relays in the RVM and in the pons (locus coeruleus) (Millan, 2002). In the case of the RVM the PAG is thought to release opioids to disinhibit a descending anti-nociceptive projection to the spinal cord (Fields, 2004, Heinricher et al., 2009). Although we found a trend towards reduced [^11^C]DPN V_T_ in the medulla (which was of a similar effect size to that seen in the PAG ~15%) this was not significant on group analysis. Given the small numbers of subjects in our study we are cautious about interpretation of this finding which will require a larger definitive study to resolve.

Although we showed evidence for DBS-evoked release of endogenous opioids we were unable to show any correlation with the acute improvement in pain or in mood with DBS. Furthermore, the systemic administration of the opioid antagonist naloxone did not produce any change in either spontaneous or evoked pain measures. This is in agreement with previous blinded DBS studies that found the analgesic effect of DBS was not blocked completely by naloxone (Young and Chambi, 1987). Given the small number of subjects our correlation analysis has limited power and therefore should be regarded as an exploratory investigation. We were also relatively cautious in our dosing of naloxone for fear of provoking uncontrolled pain flares in our patients. We used a dose (400 mcg) that is clinically recommended for the initial treatment of opioid overdose and has also been used in similar studies attempting opioid antagonist reversal of DBS (Hosobuchi et al., 1977, Young and Chambi, 1987). We estimate that this dose will have blocked around 30% of the opioid receptors based on previous naloxone displacement studies of [^11^C]DPN in man (Melichar et al., 2003). Although higher doses of naloxone (up to 10 mg) have been advocated as being necessary in studies of endogenous opioid mediated mechanisms these were found to be no more effective than lower doses in the DBS study of Young and Chambi (1987). Therefore based on the evidence from our study we have been unable to identify a role for the opioid release in the PAG in the beneficial analgesic effect of DBS but this may reflect insufficient naloxone dosing to compete with the focal release of high levels of opioid peptides.

Our pragmatic study design, of necessity, has several limitations because of the challenges of working with this patient population with severe pain and the small number of subjects; a consequence of recruiting from a scarce cohort with implanted DBS systems for de-afferentation pain, who also had to exhibit a temporally sharp on–off response to stimulation, and could tolerate a long period without stimulation during scanning. Blinding of the study participants was not feasible as they were aware that their stimulator had been switched off. However, we think it unlikely that our findings represent a placebo effect as the location of the cluster of voxels was close to the site of stimulation within the PAG – this would be a remarkably specific placebo response. Additionally, the sequence of the PET scans could not be randomised to avoid order effects, given the need to study the change in opioid binding when DBS was activated (and the constraint of a single arterial cannulation, if at all possible, which meant doing two scans on a single day). It is worth noting that we found no evidence for order effects as there was no difference in the whole brain [^11^C]DPN V_T_ between the two scan sessions and no focal change in [^11^C]DPN K_1_ or rCBF. Notwithstanding these caveats, the demonstration of changes in opioid binding in the PAG indicates the sensitivity of the PET methodology and speaks to the magnitude of the release of opioid by DBS.

Our study shows that DBS of the PAG causes a focal reduction of opioid binding in a discrete cluster consistent with the local release of endogenous opioid peptides. This release is seen in the dorsal quadrant of the PAG close to the sites of stimulation and in a region with high opioid binding. It remains to be determined whether this endogenous opioid release is playing a significant role in the production of analgesia.

## Figures and Tables

**Table 1:** Inclusion and exclusion criteria.

**Inclusion criteria**	**Exclusion criteria**
• Implanted PAG DBS system for de-afferentation pain	• Pre-existing structural brain abnormality
• Maintained improvement in pain score of >30%	• Inability to tolerate 3 h without DBS
• Presence of clear temporal analgesic response to DBS	• Contraindication to PET scanning
• Able to provide informed consent	• Unsuitability for arterial cannulation
• Able to comply with all testing protocols	• Unstable analgesic medication
	• Age <30 years
	• Pregnancy

**Table 2:** Subject demographics.

**Study ID**	**Sex**	**Age**	**Pain syndrome**	**Duration of DBS (months)**	**Electrode side**	**Target(s)**
**h00631**	F	50	Anaesthesia Dolorosa	15	Left	PAG, CM-Pf
**h00634**	M	31	Anaesthesia Dolorosa	7	Right	PAG, CM-Pf
**h00635**	F	52	Anaesthesia Dolorosa	34	Right	PAG, CM-Pf
**h00636**	M	63	Phantom Limb Pain	60	Left	PAG, VPL
**h00683**	M	50	Phantom Limb Pain	64	Right	PAG, CM-Pf

DBS – deep brain stimulation, PAG – Periaqueductal gray, CM-Pf centre median-parafasicular complex.

VPL – ventral posterolateral nucleus of the thalamus.

**Table 3:** Change in POMS_SV_ score with DBS.

**POMS**_**SV**_**subset score**	**%change with DBS active**	***P***
Anxiety	−45.0	0.001
Depression	−14.3	0.37
Anger	−11.1	0.37
Vigour	20.7	0.30
Fatigue	−48.4	0.10
Confusion	−46.7	0.08
**Total POMS**_**SV**_	−73.6	0.008

POMS_SV_**–** Profile of Mood State Shortened Version score (Shacham, 1983). (Two tailed paired *t*-test).
