# Supplementary material for: Deep brain stimulation of the periaqueductal gray releases endogenous opioids in humans
Source: Neuroimage. 2017 Feb 1;146:833–42. doi: 10.1016/j.neuroimage.2016.08.038 (PMC5312788; doi:10.1016/j.neuroimage.2016.08.038)
Supplement: Supplementary file 1 — Supplementary material [file mmc1.docx]

**Supplemental Material:**

**Study subject clinical vignettes**

*Subject H00631:*

A 51-year-old woman with Anaesthesia Dolorosa had left-sided dual DBS electrode implantation targeting PAG and CM-Pf in 2011. She presented in 2007 with a four-week history of severe right-sided facial pain extending from her ear to right forehead and retro-orbital region with radiation to maxilla and hard palate. Triggers included cold wind, cold drinks and brushing her teeth. MRI showed vascular compression of right trigeminal nerve at the root entry zone. After a diagnostic trigeminal ganglion glycerol injection the patient developed numbness but had no improvement of pain. The patient was then diagnosed with temporo-mandibular joint dysfunction and treated with an occlusal splint and liquid diet with some success. However, the facial numbness remained and a constant and burning pain developed consistent with a diagnosis of anaesthesia dolorosa. She was offered DBS as her pain was unresponsive to other treatment approaches. Dual target DBS gave a maintained improvement of 70% in pain scores (10/10 to 3/10).

*Subject H00634:*

A 33-year old man with Anaesthesia Dolorosa was implanted with dual right-sided DBS electrodes targeting PAG and CM-Pf in 2012. He had sustained multiple facial stab wounds from an assault in 2005. This fractured his zygoma with loss of facial sensation and taste on the left side. An MRI demonstrated a cyst like lesion in the left maxillary sinus, and this was surgically excised. Subsequently the patient developed severe throbbing pain, lancinating sensations and allodynia in the left V2 dermatome, leading to the diagnosis of anaesthesia dolorosa. Repeat MRI scanning did not show vascular compression of the trigeminal nerve. Subsequent treatment included infraorbital steroid and local anaesthetic injections, mirror therapy, psychological input, pain physiotherapy and extensive trials of pharmacotherapy. He became unable to work and he suffered with depression. Seven years after his original assault he underwent DBS and responded immediately with a maintained 70% improvement in his pain score (10/10 to 3/10) and he returned to working part-time.

*Subject H00635:*

A 54-year-old woman with Anaesthesia Dolorosa underwent DBS to the right PAG and CM-Pf in 2010. She originally presented in 2004 with left-sided facial pain. She was diagnosed with trigeminal neuralgia, several pharmacological therapies were tried with modest benefit (see Suppl. Table 2), and she was referred to neurosurgery where an MRI confirmed vascular compression of the trigeminal ganglion. Microvascular decompression in 2005 alleviated her symptoms for 6 weeks before the pain returned. A partial sensory rhizotomy was performed in 2006, which qualitatively altered the characteristics of her pain in keeping with the development of anaesthesia dolorosa. Her pain was predominantly lancinating in the mandibular region including teeth, tongue, eye and cheek. Cold, wind, talking, eating and tiredness exacerbated the pain, which contributed to her being unable to work and her divorce. She was referred for psychological intervention and pharmacotherapy with a modest response to opioid medication. Dual target DBS resulted in a sustained 67% reduction in pain scores (9/10 to 3/10).

*Subject H00636:*

A 64 year old man with right-sided Phantom Limb Pain underwent DBS in 2008 with electrodes implanted into the left PAG and sensory Ventro-posteriolateral (VPL) thalamus. Thirteen years earlier he was involved in a head-on road traffic accident and sustained a right-sided brachial plexus injury. Attempted plexus nerve repair was unsuccessful and an above elbow amputation was performed to reduce disability from the paralysed, insensate limb. He developed a painful phantom limb with sensations of crushed and broken fingers - similar to that experienced immediately after the accident. Application of cold stimuli to his right chest, neck or occiput produced intense referred pain in his phantom hand. Unsuccessful attempts to control his pain included local nerve injections, mirror therapy and a trial of spinal cord stimulation. Pharmacotherapy (see Suppl. Table 1), psychological input, and transcutaneous electrical nerve stimulation (TENS) all provided a limited degree of relief. His DBS surgery was successful and PAG stimulation produced a 50% reduction in pain scores (10/10 to 5/10). The VPL thalamic stimulation was ineffective and was discontinued.

*Subject H00683:*

A 49-year-old man with left-sided phantom limb pain had DBS implantation in 2008 with leads targeting right PAG and CM-Pf. He was injured in an industrial accident; sustaining a fracture dislocation of his shoulder, multiple fractures of his arm and ribs with an associated pneumothorax. Emergency trauma-surgical management included a left above-elbow-amputation, and only after the operation did he notice upper arm pain. He developed a painful phantom limb stuck in a flexed position, with aching and itching of the elbow, and a painful paraesthesia that shot into his hand when his stump was touched. Several areas of referred sensation were found on the stump. He had a past medical history of substance misuse including alcohol, smoking of crack cocaine, heroin and cannabis. Pain team interventions included psychological counselling, use of a prosthesis and mirror therapy in addition to pharmacotherapy. The DBS resulted in a 90% (10/10 to 1/10) reduction in pain scores, and at 3-year follow-up clinic he was no longer using any analgesic medications or substances of abuse.

**Supplementary Table S1 DBS lead stimulation parameters**

|  | **Stimulation Parameters** | |
| --- | --- | --- |
| **Subject** | **PAG electrode** | **CM-Pf / VPL electrode** |
| h00631 | 1V x 150uS @ 10Hz  (Monopolar: lead 2-, 3-, case+) | CM-Pf: not used during study |
| h00634 | 4·3V x 90uS @ 10Hz  (Monopolar: lead 0-,1-,2-,3-, case+) | CM-Pf: 2V x 60uS @ 150Hz  (Bipolar: lead 2+,3-)  On for study |
| h00635 | 5V x 120uS @ 5Hz  (Bipolar: lead 0-, 3+) | CM-Pf: 2·5V x 90uS @ 70Hz  (Bipolar: lead 0-, 1+)  On for study |
| h00636 | 3V x 150uS @ 5Hz  (Monopolar: lead 1-, case+) | VPL: not in use. |
| h00683 | 2·5V x 180uS @ 10hz  (Monopolar: 0-, Case +) | CM-Pf: 2·5V x 330uS @ 130Hz  (Monopolar: lead 2-, case+)  On for study |

PAG – Periaqueductal Gray, CM-Pf - Centre Median Parafascicular nucleus, VPL – ventral posterolateral nucleus of the thalamus. –ve lead = active cathode contact(s)

**Supplementary Table S2: Medication prior to DBS implantation / time of scanning**

| **Subject** | **Prior to DBS implantation** | **At time of [^11^C]DPN PET scan *** |
| --- | --- | --- |
| h00631 | Paracetamol 1g PRN  Pregabalin 200mg TDS  Venlafaxine M/R 150mg OD  Diazepam 4mg PRN  Tramadol M/R 150mg nocte  Carbamazepine 100mg 6*daily  *[Previously: Gabapentin]* | Paracetamol PRN  Pregabalin 200mg BD  Venlafaxine M/R 150mg OD  Diazepam 4mg PRN |
| h00634 | Gabapentin 700mg TDS  Buprenorphine 10mcg/hr  Lignocaine patch 5%  Tramadol 100mg PRN  Mirtazapine 30mg BD  Nortriptyline 75mg OD  *[Previously: Carbamazepine, Oxycarbamazepine, Lamotrigine, Oxycodone, intravenous/nasal lidocaine]* | No medication |
| h00635 | Morphine (MST) 30mg BD  Amitriptyline 150mg Nocte  Pregabalin 300 BD  Carbamazepine 200mg 5*day  *[Previously: Fluoxetine, Venlafaxine, Na-Valproate and Gabapentin]* | Morphine (MST) 30mg OD  Amitriptyline 150mg Nocte  Pregabalin 150mg BD |
| h00636 | Gabapentin 200mg 5*day  Pregabalin 75mg 5*daily  Co-codamol 8/500mg TT PRN  Cannabis Smoked  *[Previously: Lamotrigine, Tramadol, DF118, Amitriptyline, Carbamazepine]* | Gabapentin 500mg OD  Pregabalin 375mg OD |
| h00683 | Pregabalin 150mg BD + 75mg BD  Mirtazapine 30mg BD  Oramorph 10-20mg QDS  Morphine sulphate MR 100mg BD  *[also obtained analgesic benefit from smoking Cocaine and Heroin]* | No medication |

* Based on medication diary completed for the five days prior to PET scanning. Medication stable over the preceding 6 weeks in all subjects.
